# Herpes simplex virus reactivation in a decade-old carpal tunnel surgery scar: A case report and review of management strategies

**DOI:** 10.1016/j.jdcr.2025.09.026

**Published:** 2025-10-10

**Authors:** Yash Jani, Preet Jani, Steven Kent

**Affiliations:** a Medical College of Georgia, Augusta University, Augusta, Georgia; b Mercer University, Macon, Georgia; c Skin Care Physicians of Georgia, Macon, Georgia

**Keywords:** dermatology, dermatome C6, herpes simplex virus (HSV), HSV reactivation, surgical scar

## Introduction

Herpes simplex virus (HSV), a double-stranded DNA virus from the *Herpesviridae* family, is responsible for causing recurrent mucocutaneous infections.^1^ Following primary infection, HSV establishes latency in sensory ganglia, where it can remain dormant indefinitely. HSV reactivation occurs under various conditions, including immunosuppression, physical or emotional stress, febrile illnesses, and local trauma, causing the virus to migrate along sensory nerves to the skin or mucous membranes.^1^

Reactivated cutaneous HSV typically manifests as painful, erythematous vesicular eruptions with a punched out appearance. These outbreaks can occur in areas of recent trauma or surgery. Prompt initiation of antiviral therapy, such as valacyclovir inhibits viral replication, thereby mitigating disease severity and length of outbreak.

HSV reactivation at a previous surgical wound site is uncommon but noteworthy.

Herein, the authors describe an unusual HSV reactivation within a surgical scar. A 67-year-old female with a history of anxiety, arthritis, asthma, depression, hypercholesterolemia, hypothyroidism, and restless leg syndrome, developed a painful, blistering rash on her right radial palm, precisely at her carpal tunnel surgery scar, 10 years postoperatively. The rash corresponded to the C6 dermatome, suggesting HSV reactivation in the dorsal root ganglia of C6 spinal nerve. Initial clinical impressions suggested herpes zoster due to the dermatomal pattern;, polymerase chain reactivation (PCR) testing confirmed HSV, effectively ruling out varicella zoster virus (VZV).

While both HSV and VZV can remain latent for decades, accurate differentiation is critical. VZV reactivation typically requires higher antiviral dosing (eg, valacyclovir 1 g TID for 7-10 days) and carries a greater risk of complications such as postherpetic neuralgia. In contrast, HSV often recurs more frequently and responds to lower antiviral dosages. Therefore, establishing the correct diagnosis through PCR is essential for guiding effective treatment.

## Case presentation

A 67-year-old female presented with a 1-week history of a painful, blistering rash localized to her right radial palm, corresponding to the C6 dermatome. The rash appeared along the scar line from her carpal tunnel surgery performed 10 years prior. Prodromal symptoms included localized burning, itching, and pain confined to the right radial palm; however, systemic B-symptoms such as fever, malaise, or chills were not reported.

Her medical history included hypothyroidism, hyperlipidemia, asthma with recent corticosteroid use for exacerbation, and inflammatory arthritis under rheumatologic evaluation for possible rheumatoid arthritis. She had recently initiated methotrexate. The patient denied any recent trauma or procedures involving the right hand, and reported no prior herpetic lesions at that site.

**Fig 1:**
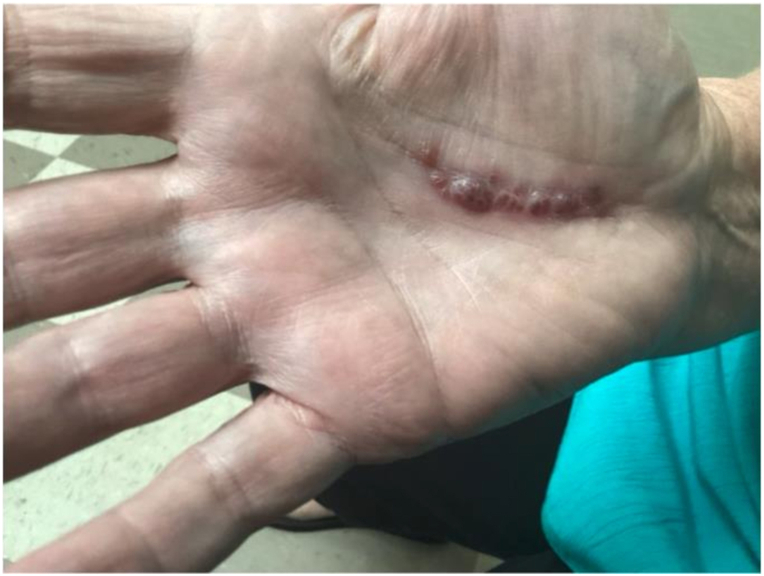
Pretreatment appearance of the blistering rash along the carpal tunnel surgery scar, showing grouped vesicles with an erythematous base.

Examination revealed grouped erythematous vesicles and erosions on an erythematous base along the surgical scar, (Fig 1).

Although the distribution roughly aligned with the C6 dermatome, it lacked the classic, broader band-like morphology typical of herpes zoster. Instead, the presentation was more consistent with a “herpeticum scariform” pattern—HSV reactivation localized to a site of previous trauma.

The patient was prescribed valacyclovir 1 gram orally 3 times daily for 7 days. Though the antiviral regimen was initiated approximately 1 week after lesion onset—outside the ideal 48-hour window—marked clinical improvement was observed. Topical antivirals were not administered.

PCR testing was ordered to confirm the diagnosis and differentiate between VZV and HSV. PCR testing confirmed presence of HSV DNA within the vesicles.

**Fig 2:**
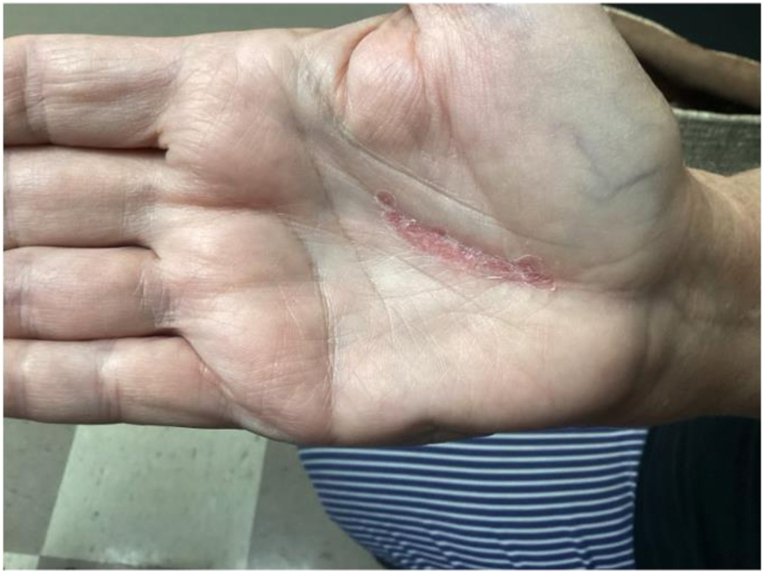
Post-treatment appearance after 1 week of valacyclovir, showing resolution of the vesicles and healing of the skin.

Follow-up showed significant improvement wit resolution of the vesicles (Fig 2). The patient was advised to continue the antiviral regimen and apply Vaseline to the affected area.

This case underscores the importance of differentiating between VZV and HSV in postoperative dermatologic presentations and highlights the unusual appearance of cutaneous HSV occurring within a prior surgical scar. HSV reactivation at a previous surgical site, specifically postcarpal tunnel surgery, is rare but notable.

## Discussion

HSV affects 60% to 90% of adults while establishing latency in sensory ganglia following primary infection. Asymptomatic primary infection occurs in up to 90% of affected individuals.^2^ Stressors like trauma, surgery, and ultraviolet light exposure, can trigger reactivation, causing the characteristic vesicular lesions.^2^

Our case is unique due to the 10-year latency period. The patient experienced HSV reactivation nearly 10 years after her initial carpal tunnel surgery, a longer duration than other reported cases. Most cases involve recent procedures, typically within weeks or a few months postoperatively. This distinction highlights the need to carefully differentiate between surgery as a predisposing factor versus a direct trigger.

Prodromal symptoms are important to consider in HSV reactivation. These often include local burning, tingling, or pruritus, and may sometimes extend to systemic “B-symptoms” such as fever, malaise, or chills. In our patient, the prodrome was limited to localized burning and pain at the surgical scar site, with no constitutional symptoms reported. This pattern is consistent with a localized HSV reactivation rather than a systemic disease.

This case illustrates the concept of “locus minoris resistentiae”, in which prior tissue damage—such as surgical manipulation—creates a site of decreased resistance. Here, the prior carpal tunnel surgery served as a predisposing factor, not the direct trigger. The likely trigger was recent systemic immunosuppression, including corticosteroids for asthma and methotrexate for inflammatory arthritis. Distinguishing between locus minoris resistentiae (a vulnerable site) and a true triggering event is critical for proper clinical interpretation and management planning. If surgery itself is the trigger (ie, viral reactivation occurs during or immediately after the procedure), prophylactic antivirals may be considered in the perioperative period. However, when surgery merely predisposes the site and reactivation occurs years later due to systemic immunologic shifts, long-term management must focus on monitoring immunosuppressive states and recurrence risk.

Beyond local surgical vulnerability, systemic immunosuppression represents a critical trigger for HSV reactivation. Methotrexate, widely used in autoimmune diseases, has been increasingly linked to atypical or severe HSV presentations. One report described a patient with dermatomyositis on long-term methotrexate and low-dose corticosteroids who developed painful oral stomatitis with fever and pancytopenia; while initially mistaken for methotrexate toxicity, PCR confirmed HSV-1 reactivation, and acyclovir led to rapid resolution.^3^ Another case involved a patient with rheumatoid arthritis receiving methotrexate and tofacitinib who developed hemorrhagic crusting of the lips and widespread oral erosions; histopathology confirmed HSV infection, which improved only after antivirals were initiated.^4^ A third report described disseminated cutaneous HSV-1 infection in a patient with rheumatoid arthritis on methotrexate, corticosteroids, and infliximab, requiring high-dose acyclovir and prolonged prophylaxis.^5^

Taken together, these cases demonstrate that methotrexate can facilitate HSV reactivation ranging from localized mucocutaneous disease to disseminated systemic infection (Table I). In contrast to trauma-triggered cases summarized in Tables II and III, which were driven by acute surgical injury, methotrexate-associated reactivations represent a distinct mechanism involving systemic impairment of immune surveillance. Our case highlights the intersection of both processes: a locus minoris resistentiae established by prior surgery, combined with methotrexate-related immunosuppression as the proximate trigger for HSV reactivation.

**Table I:** HSV reactivation in the context of methotrexate and immunosuppressive therapy

Study/case report	Virus type	Clinical setting	Immunosuppressive therapy	Symptoms	Diagnostic method	Treatment	Outcome
Our case	HSV1	Surgical scar, right radial palm, 10 y postcarpal tunnel surgery	Corticosteroids for asthma + Methotrexate for inflammatory arthritis	Localized burning, pain, grouped vesicles along scar (C6 distribution)	PCR of swab	Valacyclovir 1 g TID × 7 d	Resolution within 1 wk
Akagi et al (2021)^3^	HSV-1	Dermatomyositis	Methotrexate 12 mg/wk + Prednisolone 5 mg/d	Severe oral stomatitis, fever, pancytopenia	PCR of oral lesions, serology	Acyclovir	Rapid resolution
Alghamdi et al (2023)^4^	HSV (unspecified)	Rheumatoid arthritis	Methotrexate + Tofacitinib	Hemorrhagic lip crusting, painful oral erosions	Histopathology	Acyclovir	Resolution within 1 wk
Justice et al (2008)^5^	HSV-1	Rheumatoid arthritis	Methotrexate + Prednisolone + Infliximab	Disseminated vesicular rash, fever	PCR of vesicle fluid	High-dose acyclovir, prophylactic suppression	Recovery; no recurrence on prophylaxis

*HSV*, Herpes simplex virus; *PCR*, polymerase chain reactivation.

**Table II:** Comparison of published cases of HSV at surgical sites

Study/case report	Virus type	Infection site	Time postsurgery	Symptoms	Diagnostic method	Treatment	Outcome
Our case	HSV1	Right radial palm	10 y	Redness, pain, vesicles	PCR of swab	Valtrex 1 g TID × 1 wk	Resolution of symptoms
Parys et al (2013)	HSV1	Free radial forearm	3 d	Dark color, vesicles	PCR of swab, microscopy, culture	Acyclovir 400 mg 5×/d	Complete recovery of the flap
Dzubow (1989) case #1	HSV1	Cheeks and chin	2 wk	Erythema, erosion	Viral culture	Acyclovir 200 mg 5×/d	Excellent cosmetic result, no scarring
Dzubow (1989) case #2	HSV1	Left chin	6 d	Erythema, exudation	Viral culture	Acyclovir 200 mg 5×/d	Central portion of scar atrophic and depressed
Dzubow (1989) case #3	HSV1	Right upper lip	6 d	Erythema, edema, vesicles	Tzanck prep, viral culture	Acyclovir 200 mg 5×/d	Partial flap necrosis, shiny atrophic scar
Hernández et al (2011)	HSV1	Oral mucosa	Days to wk	Vesicles, graft failure	PCR of swab, viral culture	Acyclovir 200 mg 5×/d	Graft failure
Alexander and Wismer (2003)^6^	HSV1	Total hip arthroplasty site	Days to wk	Vesicles, superficial wound infection	Viral culture, clinical presentation	Acyclovir	Superficial wound infection resolved

*HSV*, Herpes simplex virus; *PCR*, polymerase chain reactivation.

**Table III:** Comparison of published cases of VZV at surgical sites

Study/case report	Virus type	Infection site	Time postsurgery	Symptoms	Diagnostic method	Treatment	Outcome
Nikkels and Piérard (1999) case #1	VZV	Right T5 dermatome	16 d	Zosteriform eruption centered on surgical scar	Tzanck smear, skin biopsy	Intravenous acyclovir 10 mg/kg q8h	Patient died from complications of acute respiratory distress syndrome
Nikkels and Piérard (1999) case #2	VZV	Left T2 dermatome	16 d	Vesicular lesions on surgical scar	Immunohistochemistry, skin biopsy	Intravenous acyclovir 10 mg/kg q8h	Lesions resolved in 10 d
Choi et al (2012)	VZV	Subciliary incision scar	10 d	Tingling, swelling, vesicles	Clinical presentation	Acyclovir 800 mg 5×/d for 1 wk, prednisone 200 mg daily for 5 d	Quick and uneventful recovery over 2 wk
Massad et al (2004)	VZV	Right T3-T4 dermatomes	1 d	Pain, swelling, erythema, vesicles	Clinical presentation	Valacyclovir 500 mg BID for 10 d	Gradual pain subsiding over 2 mo
Muesse et al (2012)	VZV	T6 dermatome	1 wk	Pain, erythema, vesicles	Clinical presentation	Valacyclovir for 10 d	Rapid recovery, no postherpetic neuralgia

*VZV*, Varicella zoster virus.

Another important diagnostic consideration was primary HSV infection, particularly because no prior outbreaks were documented at this site. Primary HSV typically presents with more widespread cutaneous involvement, systemic prodromal symptoms such as fever, malaise, or lymphadenopathy, and prolonged lesion persistence before resolution. In contrast, our patient’s disease was confined to a previously traumatized site, lacked systemic prodrome, and resolved rapidly with standard antiviral therapy. These features collectively favored reactivation in an immunocompromised host rather than primary infection, though including primary HSV in the differential remains essential, particularly in patients with altered immunity who may present atypically.

This interpretation is further supported by mechanistic considerations. Local vascular integrity and immune surveillance strongly influence HSV replication and lesion severity. Histopathologic hallmarks include balloon degeneration, acantholysis, dyskeratosis, and keratinocyte necrosis.^1^ Vascular endothelium vulnerability depends on skin vascular health.^1^ In the present case, erythematous vesicles and erosions along the decade-old carpal tunnel surgery scar are consistent with localized reactivation at a site of diminished resistance, rather than the broader and more severe pattern typical of primary infection.

A critical aspect was differentiating between HSV and VZV, both presenting with similar dermatomal vesicular rashes. Although the rash in this patient approximated the C6 dermatome, the morphology and localization along a surgical scar were more consistent with HSV. PCR testing, which is extremely sensitive (96%) and specific (99%), confirmed HSV.^6^ This distinction is essential because VZV requires higher-dose antivirals and carries a greater risk of chronic neuropathic complications, while HSV is managed with lower-dose regimens and tends to recur more frequently.

The decade-long latency period before HSV reactivation challenges the current understanding that reactivation is typically linked to recent trauma or acute immunosuppression. It raises important questions about factors contributing to delayed reactivation. The patient’s comorbidities—particularly asthma and inflammatory arthritis requiring chronic immunomodulatory therapy—may have gradually altered immune surveillance over time, subtly promoting HSV reactivation at a site of previous tissue injury.

The anatomical localization of the eruption is also notable. Carpal tunnel surgery involves manipulation of the median nerve and surrounding soft tissue, which may have created localized neural susceptibility. While dorsal root ganglia are known reservoirs for HSV latency, precise reactivation in the C6 dermatome—10 years after nerve manipulation—suggests a complex interplay between neuroanatomy, inflammation, and viral pathogenesis.

Although prompt antiviral therapy was initiated outside the typical 48-hour window, it led to lesion resolution and prevented complications such as secondary bacterial infection or chronic neuropathic pain. This case reinforces the importance of maintaining a high index of suspicion for HSV in atypical presentations and initiating treatment promptly once confirmed.

## Conclusion

This case highlights a rare presentation of HSV reactivation occurring a decade after carpal tunnel surgery. It underscores the importance of distinguishing surgery as a predisposing factor (locus minoris resistentiae) rather than a direct trigger, and it emphasizes the role of systemic immunosuppressive therapy in viral reactivation. Clinicians should consider HSV in the differential diagnosis of delayed vesicular eruptions near surgical sites, especially in immunocompromised patients. Further research is warranted to explore the long-term interplay between tissue damage, immune modulation, and neural latency in HSV pathogenesis.

## Conflicts of interest

None disclosed.
